# PCT, IL-6, and IL-10 facilitate early diagnosis and pathogen classifications in bloodstream infection

**DOI:** 10.1186/s12941-023-00653-4

**Published:** 2023-11-20

**Authors:** Xianggui Yang, Jun Zeng, Xuejing Yu, Zhenguo Wang, Dan Wang, Qin Zhou, Tingting Bai, Ying Xu

**Affiliations:** 1 Department of Laboratory Medicine, Clinical Medical College and the First Affiliated Hospital of Chengdu Medical College, Chengdu, Sichuan China; 2 Division of Pulmonary and Critical Care Medicine, Clinical Medical College and the First Affiliated Hospital of Chengdu Medical College, Chengdu, Sichuan China; 3 Department of Internal Medicine, Division of Cardiology, University of Texas Southwestern Medical Center, Dallas, TX USA; 4 Department of Stomatology, Clinical Medical College and the First Affiliated Hospital of Chengdu Medical College, Chengdu, Sichuan China

**Keywords:** Procalcitonin, Interleukin-6, Interleukin-10, Bloodstream infection, Pathogens classification

## Abstract

**Background:**

In the diagnosis of bloodstream infection (BSI), various inflammatory markers such as C-reactive protein (CRP), procalcitonin (PCT), interleukins (IL), white blood cell count (WBC), neutrophil percentage (NE%), platelet count (PLT), and erythrocyte sedimentation rate (ESR) have been extensively utilized. However, their specific roles in distinguishing BSI from local bacterial infection (LBI) and in classifying BSI pathogens remain uncertain.

**Methods:**

A historical cohort study was conducted, involving the enrollment of 505 patients with BSI and 102 patients with LBI. To validate the reliability of the clinical data obtained from this cohort, mouse models of BSI were utilized.

**Results:**

Our findings revealed that patients with BSI had significantly higher levels of inflammatory markers, including CRP, PCT, IL-6, IL-10, WBC, NE%, and ESR, compared to those with LBI (p < 0.05). The receiver operating characteristic (ROC) curve analysis demonstrated that CRP, PCT, IL-6, IL-10, ESR and NE% exhibited excellent diagnostic efficacy for BSI. Additionally, we observed significant differences in CRP, PCT, IL-6, and IL-10 levels between patients with BSI caused by Gram-positive bacteria (GP-BSI) and Gram-negative bacteria (GN-BSI), but no significant variations were found among specific bacterial species. Furthermore, our study also found that CRP, PCT, and IL-10 have good discriminatory ability for vancomycin-resistant *Enterococcus* (VRE), but they show no significant diagnostic efficacy for other multidrug-resistant organisms (MDROs) such as carbapenem-resistant *Enterobacteriaceae* (CRE), carbapenem-resistant *Pseudomonas aeruginosa* (CRPA), and methicillin-resistant *Staphylococcus aureus* (MRSA). In our mouse model experiments, we observed a remarkable increase in PCT, IL-6, and IL-10 levels in mice with GN-BSI compared to those with GP-BSI.

**Conclusion:**

Our study has confirmed that PCT, IL-6, and IL-10 are efficient biomarkers for distinguishing between BSI and LBI. Furthermore, they can be utilized to classify BSI pathogens and differentiate between VRE and vancomycin-susceptible *Enterococcus*. These findings are extremely valuable for clinicians as they enable timely initiation of empiric antibiotic therapies and ultimately lead to improved clinical outcomes for patients with BSI.

**Supplementary Information:**

The online version contains supplementary material available at 10.1186/s12941-023-00653-4.

## Background

Bloodstream infection (BSI) is a thorny clinical problem with a high mortality rate in current days, it is threatening especially in patients with diabetes, liver failure, leukemia, respiratory failure, and severe pneumonia [1]. Mounting evidence shows that around 28.3–41.1% of BSI patients lost their lives in North America and Europe from 2005 to 2010, and the primary cause of death is multiple organ failure [2].

BSI is generally considered as a two-phase disease, nevertheless, both phases can manifest concurrently [3, 4]. The initial phase is a hyperinflammatory state known as the “cytokine storm”. During this phase, the innate immune system releases excessive inflammatory molecules, leading to tissue damage [3]. Subsequently, the immune response dampens, resulting in a hypo-inflammatory state. In this condition, the immune system experiences exhaustion, leading to the death of cells from both lymphoid and myeloid lineages [5, 6]. Consequently, patients become immunocompromised, leaving them vulnerable to infections and other health challenges.

When necrotic tissue and microbes are present, they release detrimental substances into the body. These substances include damage-associated molecular patterns (DAMPs) and pathogen-associated molecular patterns (PAMPs) [7]. The release of these detrimental substances activates pattern recognition receptors (PRRs) expressed in innate immune cells, such as toll-like receptors (TLRs) [8]. The activation of these receptors further stimulates rapid proliferation of innate immune cells like macrophages, dendritic cells, and neutrophils, accompanied by the release of numerous cytokines, such as IL-1β, IL-2, IL-6, TNF-α, IL-8 and IL-10 [9]. Subsequently, the adaptive immune system is triggered, leading to the activation of T helper cells and cytotoxic T cells. This process results in the differentiation and proliferation of these cells, generating a remarkably targeted and specific adaptive immune response [10].

Currently, blood culture is recognized as the “gold standard” for diagnosing BSI. However, it is time-consuming and usually takes 3–5 days to obtain a confirmed diagnosis [11]. Moreover, the sensitivity of blood culture can be significantly reduced due to the slow growth rate of fastidious pathogens or prior antibiotic use before blood collection [12]. Given the swift progression and high mortality rate associated with BSI, the timely administration of appropriate antibiotics to affected patients is vital for reducing mortality and enhancing prognosis [12]. This necessitates clinicians to administer empirical broad-spectrum antibiotics prior to receiving definitive results from blood cultures [13]. However, this approach can be a double-edged sword, potentially fostering the emergence of multidrug-resistant pathogens, thereby posing serious risks to patients [14]. As treatment strategies differ significantly between BSIs caused by gram-negative bacteria (GN-BSI) and gram-positive bacteria (GP-BSI), the ability to classify these pathogens in cases of BSI becomes immensely valuable in guiding early, precise, and effective therapeutic interventions [12].

Numerous inflammation-related biomarkers have been documented, including white blood cell count (WBC), neutrophils percentage (NE%), C-reactive protein (CRP), erythrocyte sedimentation rate (ESR), interleukins (IL-6 and IL-10), procalcitonin (PCT), platelet count (PLT), vascular endothelial growth factor (VEGF), tumor necrosis factor-α (TNF-α), macrophage inflammatory protein-1β (MIP-1β), IL-2, and IL-8 [15]. Among these markers, WBC, CRP, NE%, PLT, ESR, PCT, IL-6, and IL-10 have been identified as potential diagnostic biomarkers for BSI [1]. For instance, the combination of PCT and CRP has shown promise in diagnosing BSI, particularly for sepsis [16, 17], while IL-6 has proven useful in distinguishing sepsis from non-infectious systemic inflammatory response syndrome [18]. However, despite their diagnostic potential for BSI, there is limited research investigating their early use in distinguishing BSI from local bacterial infections (LBI) or identifying the classification of BSI bacteria (Gram-positive/Gram-negative), especially in adult patients without underlying conditions such as blood disorders, autoimmune disorders, and tumors.

In this study, we found that CRP, PCT, IL-6, IL-10, ESR, and NE% were useful biomarkers to differentiate BSI from LBI. Moreover, PCT, IL-6, and IL-10 levels were significantly different in patients with GP-BSI and GN-BSI, indicating that PCT, IL-6, and IL-10 can be applied to distinguish GN-BSI from GP-BSI.

## Methods

### The design for the cohort study

In retrospective cohort investigation, patient data encompassing cases of both BSI and LBI were meticulously compiled at the First Affiliated Hospital of Chengdu Medical College between January 2017 and April 2023. This medical center, situated in Chengdu, China, boasts a comprehensive tertiary setup with a bed capacity of 1800. To establish the presence of infectious pathogens, two blood culture sets were procured via blood cultures from distinct bodily locations. Examples of these pairings include the left and right elbow veins, upper and lower limb veins, or peripheral and central veins. Each blood culture set consists of 2 vials, one vial is allocated for aerobic culture, while the other is designated for anaerobic culture. This methodology aimed to differentiate authentic bloodstream infections, wherein both specimens yield positive results for the same organism.

For patients diagnosed with BSI, the inclusion criteria encompassed: (1) age exceeding 18 years; (2) evident signs of infection, such as fever (≥ 38 ℃) or hypothermia (≤ 36 ℃) accompanied by chills; (3) affirmation of “two blood culture sets from bilateral sites” yielding positive cultures for corresponding pathogenic bacteria; (4) adherence to Sepsis-3 criteria [19]. Exclusion criteria consisted of: (1) blood disorders, including leukemia, immune thrombocytopenia, thalassemia, and multiple myeloma; (2) malignancies such as liver, gastric, lung, lymphatic, and ovarian cancers; (3) autoimmune ailments like systemic lupus erythematosus and vasculitis; (4) compromised liver and spleen function, including conditions like cirrhosis, fatty liver, and hypersplenism; (5) administration of antibiotic treatment within 3 days preceding blood cultures; (6) utilization of antiplatelet agents, granulocyte-boosting agents, hemostatic agents, or blood transfusion therapy (comprising platelets, red blood cells, or plasma) within 7 days before blood cultures and inflammation biomarker assessment; (7) viral infections, encompassing influenza, hepatitis viruses, enteroviruses, coxsackie virus, measles virus, Epstein-Barr virus, and human immunodeficiency virus.

**Fig. 1:**
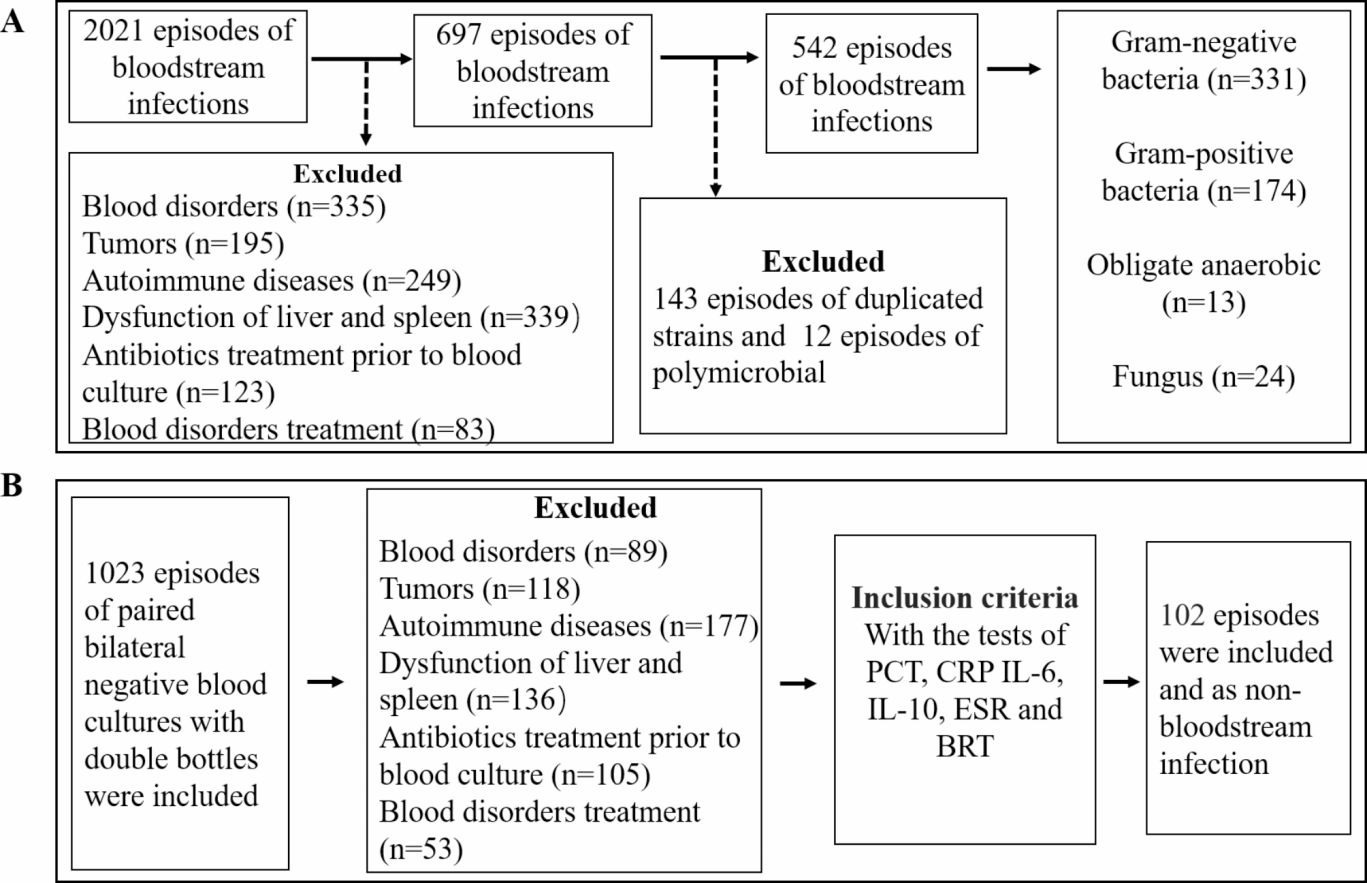
Flowchart illustrating the episode selection process. (**A**) Selection process of patients with bloodstream infection. (**B**) Selection process of patients with local bacterial infection. LBI, local bacterial infection; PCT, procalcitonin; CRP, C-reactive protein; IL-6, iinterleukin-6; IL-10, iinterleukin-10; ESR, erythrocyte sedimentation rate; BRT, blood routine test

Conversely, for patients diagnosed with LBI, inclusion criteria included: (1) age above 18 years; (2) negative results from “two blood culture sets from bilateral sites”; (3) inability to ascertain sepsis or BSI through assessments by a minimum of two experienced clinicians [19];4) positive cultures of non-blood specimens (such as urine, wound secretions, abscesses, bronchoalveolar lavage fluid, and sputum) indicating the presence of corresponding pathogenic bacteria; 5) availability of PCT, CRP, IL-6, IL-10, ESR, and blood routine test data. The exclusion criteria were: (1) administration of antibiotic therapy within 3 days prior to culturing non-blood specimens; (2) other exclusion criteria are the same as those for patients with BSI. The schematic representation of the study design is depicted in Fig. 1. Ultimately, a total of 542 BSI patients and 102 LBI patients were included in this retrospective cohort analysis.

Informed consent was waived due to the anonymized nature of clinical data from all patients, aligning with the guidelines stipulated by the Scientific Research Ethics Committee of the Institutional Review Board (IRB) at the Clinical Medical College and the First Affiliated Hospital of Chengdu Medical College. The confidentiality and safeguarding of the privacy and clinical data of all patients were stringently upheld. The entire study adhered to the principles of the Helsinki Declaration.

### Collection of clinical patient data

Comprehensive data were documented, encompassing essential aspects such as age, gender, primary diagnosis, underlying diseases, blood culture outcomes, blood routine parameters (including WBC, NE%, and PLT), CRP levels, PCT levels, as well IL-6 andIL-10 levels. The equipment employed for assessing these inflammatory biomarkers was as follows: blood culture (model: Bact/ALERT 3D-120, BioMerieux, Marcy l’Etoile, France); blood routine (model: XN-9000, Sysmex, Shanghai, China); CRP (7600, Hitachi, Ltd., Chiyoda-ku, Japan); PCT (model: cobase e-602, Roche, Switzerland); IL-6 and IL-10 (model: DxFLEX, Beckman Coulter, USA). The reagents employed for assessing these inflammatory biomarkers was as follows: blood culture (410,851/2, BioMerieux, Marcy l’Etoile, France); blood routine (984-1721-6, Sysmex, Shanghai, China); CRP (0316051, Maccura, Chengdu, China); PCT (09318712190, Roche, Switzerland); IL-6 and IL-10 (BNCBA002, Raisecare Biological Technology, Qingdao, China).

### Statistical analysis based on clinical data in cohort study

To investigate the diagnostic potential of diverse inflammatory biomarkers in identifying cases of BSI, the primary focus of this study revolved around two key groups: the BSI group and the LBI group. Additionally, for a more refined understanding of the categorization of pathogenic bacteria within BSI cases (G + or G-), the BSI group was further segregated into two distinct subgroups: GP-BSI and GN-BSI. Demographic and clinical variables, encompassing age, gender, inpatient departments, and primary diagnosis, underwent analysis using the *X*^2^ test or Mann-Whitney U test. The diagnostic accuracy of the different inflammatory biomarkers was assessed using ROC curves and corresponding AUC values, with DeLong’s test applied for analysis. Statistical analysis was performed using Prism V.6 software by GraphPad, and statistical significance was determined at a significance level of *P* < 0.05.

### Preparation of murine BSI models

To investigate the dynamic fluctuations of the aforementioned biomarkers, we established reliable BSI mouse models through intravenous injection of Gram-positive (*Staphylococcus aureus*) and Gram-negative (*Escherichia coli*) bacterial strains. The process began by determining half of the lethal dose 50 (LD50) for both standard strains (*Staphylococcus aureus* ATCC 25,923 and *Escherichia coli* ATCC 25,922) in mice. In summary, the two standard bacterial strains were cultivated in LB liquid medium at 37 °C for 16–18 h, after which the bacteria were harvested via centrifugation. Subsequently, a 4.0 McFarland unit (MCF) bacterial suspension was prepared according to the Maxwell turbidity method. Following this, the bacterial suspension underwent sequential 10-fold dilutions for a total of 6 dilutions. Finally, suspensions with distinct concentrations were injected into specific-pathogen-free (SPF) BALB/c mice aged 6–8 weeks, weighing between 25 and 30 g, via the tail vein with an injection volume of 0.1 mL/10 g (body weight). Over the ensuing 7 days, mouse mortality, behavioral changes, and body weight shifts were recorded. The LD50 was deduced using the Karber method [20], revealing LD50 values of 6.4 × 10^8^ CFU/mL for *Staphylococcus aureus* ATCC 25,923 and 1.2 × 10^8^ CFU/mL for *Escherichia coli* ATCC 25,922 in mice. Subsequently, to ensure the survivability of mice during infection, half of the determined LD50 was chosen as the injection dosage for constructing the BSI models. The mice were administered 1/2 LD50 of *Staphylococcus aureus* ATCC 25,923 or *Escherichia coli* ATCC 25,922 via the tail vein, while the control group received only the vehicle (normal saline). Blood samples were collected through a retro-orbital approach at specific time points post-inoculation. Anesthesia with isoflurane was utilized, and blood was collected by gently inserting a pipette tip (Fisher Scientific, Pittsburgh, PA) into the medial canthus of the eye, in accordance with established methodologies [21, 22]. After blood collection, gentle pressure was applied with a clean gauze to the eyeball to prevent further bleeding. Prior to sacrifice, additional retro-orbital blood samples were drawn from the contralateral eye, followed by immediate CO^2^ euthanasia. Serum was obtained by centrifugation at 1500 g/4℃/10min and stored at -80℃ for subsequent assessment of inflammatory markers. A total of 102 mice were utilized in this study, classified into three distinct groups: the normal saline group (control group), the *Staphylococcus aureus* infection group (GP-BSI group), and the *Escherichia coli* infection group (GN-BSI group). At each designated time point, each group comprised 5 or 6 mice. To ensure the integrity of data and minimize any potential discomfort to the animals, all animal experiments underwent repeated iterations for validation. If the outcomes from both rounds of experimentation concurred, the experiments were deemed conclusive. In cases where discrepancies emerged between the results of the two batches, a third round of experiments would be conducted.

The animal study was reviewed and approved by the the Animal Ethics Committee of the Clinical Medical College and the First Affiliated Hospital of Chengdu Medical College prior to the study. All methods were carried out in accordance with relevant guidelines and regulations.

### Measurement of CRP, PCT, IL-6, and IL-10 in mice serum

The quantification of CRP, PCT, IL-6, and IL-10 levels in murine serum was executed using the enzyme-linked immunosorbent assay (ELISA) technique, adhering to the respective guidelines provided by the manufacturers. The ELISA kits utilized were as follows: Mouse CRP (C-Reactive Protein) ELISA Kit (D721027-0096), Mouse PCT (Procalcitonin) ELISA Kit (D721169-0096), Mouse IL-6 (Interleukin 6) ELISA Kit (D721022-0096), and Mouse IL-10 (Interleukin 10) ELISA Kit (D721023-0096). All kits were purchased from Sanggon Biotechnology Co., Ltd (Sangon Biotech, Shanghai, China).

### Statistical analysis of murine data

The comparison of quantitative data within two groups was conducted using the two-tailed Student t-test and the Mann-Whitney U test. For quantitative data encompassing three or more groups, the Analysis of Variance (ANOVA) test was employed to assess differences. All statistical analyses were performed utilizing Prism V.6 software from GraphPad, and statistical significance was deemed achieved when *P* < 0.05.

## Results

### The baseline of clinical parameters

Accumulating evidence has shown that levels of inflammatory biomarkers, including WBC, CRP, NE%, ESR, PCT, IL-6, and IL-10, were significantly higher in patients with bacterial infection than those with non-bacterial infection [15]. However, scant research has addressed the utility of these inflammatory biomarkers in distinguishing between different types of infections, such as bloodstream infections BSI and LBI, as well as classifying pathogenic bacteria into Gram-positive (G+) or Gram-negative (G-) strains. To address this knowledge gap, we conducted a cohort study where patients were segregated into BSI and LBI groups. Within the BSI group, clinical data were collected from a total of 2021 patients. After rigorous screening based on predefined inclusion and exclusion criteria, 542 patients with mono-bacterial infections were enrolled, comprising 331 cases of Gram-negative bacterial infections, 174 cases of Gram-positive bacterial infections, 13 cases of anaerobic bacterial infections, and 24 cases of fungal infections. Due to the limited occurrence of anaerobic and fungal infections in our study, we focused our analysis solely on the 331 cases of Gram-negative and 174 cases of Gram-positive bacterial infections in the BSI group (Fig. 1A). The strain composition isolated from blood cultures is depicted in Table 1, indicating that among Gram-negative infections, *Escherichia coli* (n = 181, 54.68%) and *Klebsiella pneumoniae* (n = 67, 20.24%) were the two dominant bacterial species. Among Gram-positive bacteria, the major strains were *Staphylococcus aureus* (n = 69, 39.66%), *Enterococcus* spp. (including *Enterococcus faecalis* and *Enterococcus faecium*, n = 38, 21.84%), and *Streptococcus* spp. (n = 34, 19.54%). In the LBI group, clinical data from 1023 patients were meticulously recorded and subjected to thorough screening using predefined inclusion and exclusion criteria (Fig. 1B). Ultimately, 102 patients were included in this study. Notably, there were no significant statistical differences in age, gender, primary diagnosis, or inpatient departments between the BSI and LBI groups. Concerning clinical symptoms, the incidence of fever and chills was higher in the BSI group compared to the LBI group, although there was no statistically significant difference in the incidence of shock between the two groups (Table S1).

**Table 1:** Microbiological characteristics in patients with BSI

G- group	N (%)	G + group	N (%)	Obligate anaerobic	N (%)	Fungi	N (%)
*Escherichia coli*	181 (54.68%)	*Staphylococcus aureus*	69 (39.66%)	*Bacteroides fragilis*	7 (53.85%)	*Candida albicans*	9 (37.50%)
*Klebsiella pneumoniae*	67(20.24%)	*Staphylococcus hominis*	14 (8.05%)	Others	6 (46.15%)	*Candida glabrata*	5 (20.83%)
*Pseudomonas aeruginosa*	16(4.83%)	*Enterococcus faecalis*	13 (7.47%)			*Candida parapsilosis*	4 (16.67%)
*Enterobacter cloacae* complex	14 (4.23%)	*Enterococcus faecium*	25 (14.37%)			Others	6 (25.00%)
*Proteus mirabilis*	12 (3.63%)	*Streptococcus spp.*	34 (19.54%)				
*Klebsiella oxytoca*	11 (3.32%)	Others	19 (10.92%)				
Others	30(9.06%)						
Total	331(100%)	Total	174 (100%)	Total	13 (100%)	Total	24 (100%)

BSI, bloodstream infection; G-, Gram-negative; G+, Gram-positive

### Value of diagnostic and predictive capacities of CRP, PCT, IL-6, IL-10, ESR, and NE% in identifying BSI

As shown in Table S2, the BSI group exhibited significantly higher levels of CRP, PCT, IL-6, ESR, WBC, and NE% compared to the LBI group (p < 0.0001). Additionally, the IL-10 levels in the BSI group were higher than those in the LBI group (p = 0.008). Conversely, BSI patients had lower serum levels of PLT compared to LBI patients (p = 0.0254). These findings underscore the crucial roles of these biomarkers in distinguishing between BSI and LBI.

Subsequently, ROC analysis was employed to assess the efficacy of these biomarkers in differentiating BSI from LBI. As depicted in Fig. S1, PCT, CRP, IL-6, IL-10, NE%, and ESR demonstrated excellent diagnostic potential for BSI, while WBC and PLT exhibited relatively weaker diagnostic power. Optimal cut-off value analysis indicated that ESR, PCT, IL-6, and IL-10 exhibited high sensitivity and specificity in diagnosing BSI (Table S3). Remarkably, ESR, previously considered a non-specific biomarker for BSI [23], proved to be a prominent diagnostic indicator in our study, with an AUC of 0.9249, along with high specificity (91.18%) and sensitivity (76.39%). All together these results demonstrate that CRP, PCT, IL-6, IL-10, ESR, and NE% are valuable biomarkers for diagnosing BSI.

### PCT, IL-6, and IL-10 are valuable biomarkers to distinguish GP-BSI from GN-BSI

BSI pose an immense global burden, with an alarmingly high mortality rate and significant medical expenses. Timely administration of appropriate antibiotic therapies can substantially improve the survival rate and prognosis of BSI patients. However, the inappropriate and irrational use of antibiotics can lead to unfavorable outcomes, such as the emergence of multidrug-resistant organisms. Hence, the accurate identification of potential pathogens is crucial to facilitate early and targeted antibiotic therapies for BSI patients. In this study, we sought to investigate the roles of specific biomarkers, including CRP, PCT, IL-6, IL-10, ESR, WBC, NE%, and PLT, in determining the classifications of BSI pathogens. First of all ,we categorized patients with BSI into two groups based on the Gram staining characteristics of the pathogenic bacteria, namely GP-BSI and GN-BSI. Analysis of demographic characteristics revealed no significant statistical differences in terms of gender, age, primary diagnosis, and inpatient departments (internal medicine, surgery, and ICU) between these two groups (Table 2). Regarding clinical symptoms, the incidence of fever and chills was significantly higher in the GN-BSI group compared to the GP-BSI group, whereas there was no significant difference in the incidence of shock between the two groups (Table 2).

**Table 2:** Baseline characteristics of patients with BSI

Variable	GN-BSI (n = 331)	GP-BSI (n = 174)	*P* value
Age, mean ± SD	62.79 ± 16.46	62.25 ± 15.45	0.7228
Gender(male/female)	178/153	89/85	0.5741
**Primary diagnoses, n (%)**			
Diabetes mellitus	25 (7.55)	13 (7.47)	0.9989
Traumatic disease	25 (7.55)	16 (9.20)	0.6073
Cholecystitis	19 (5.74)	12 (6.90)	0.6969
Pneumonia	27 (8.16)	16 (9.20)	0.738
Cardiopathy	7 (2.11)	5 (2.87)	0.7595
Peritonitis	23 (6.95)	14 (8.05)	0.7199
Urinary tract infection	31 (9.37)	14 (8.05)	0.7428
Appendicitis	6 (1.81)	5 (2.87)	0.524
Chronic lung disease	21 (6.34)	5 (2.87)	0.1363
Hypertension	9 (2.72)	7 (4.02)	0.4326
Others	138 (41.69)	67 (38.51)	
**Clinical symptoms, n (%)**			
Fever	247 (74.62)	97 (55.75)	0.0055
Shock	37 (11.18)	13 (7.47)	0.271
Chills	157 (47.43)	54 (31.03)	0.0056
**Ward of hospitalization**			
Medical	191 (57.70)	99 (56.90)	0.7797
Surgical	112 (33.84)	57 (32.76)	
Intensive care unit	28 (8.46)	18 (10.34)	

BSI, bloodstream infection; GN-BSI, bloodstream infection caused by Gram-negative bacteria; GP-BSI, bloodstream infection caused by Gram-positive bacteria; SD, standard deviation

**Fig. 2:**
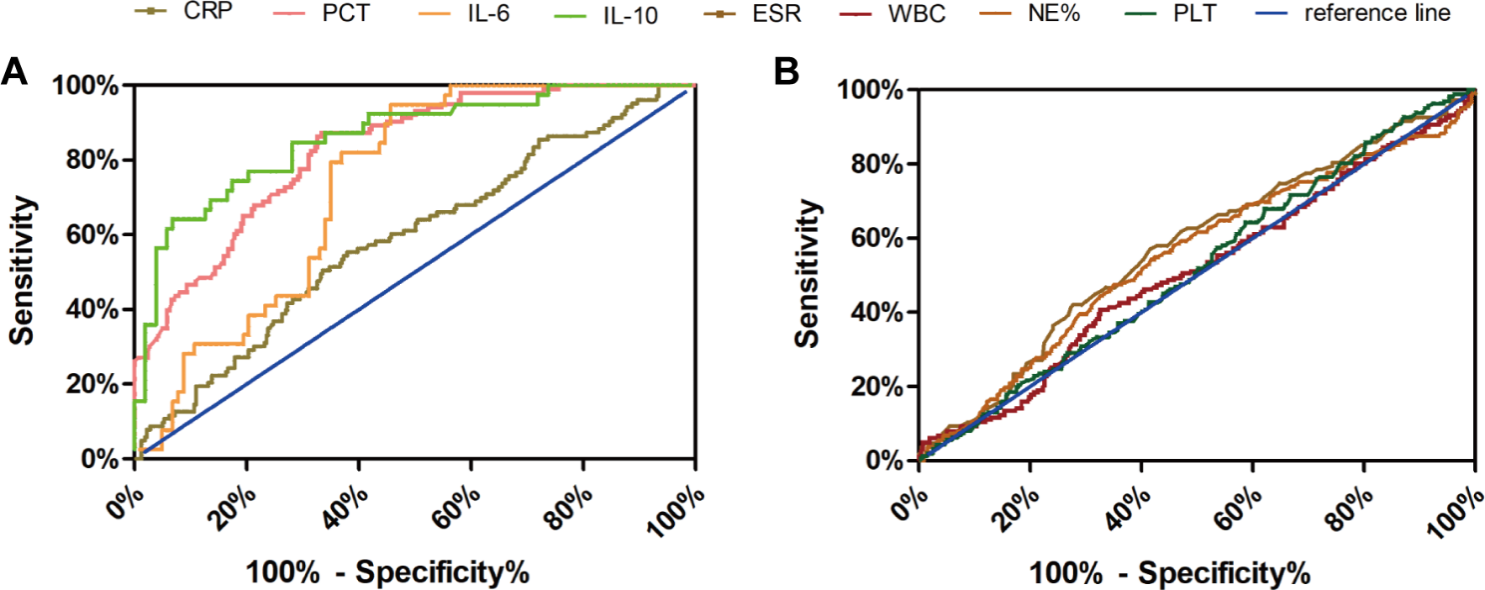
The receiver operating characteristic curves analysis applied to test discriminative performance of inflammatory biomarkers to predict GP-BSI, (**A**) CRP, PCT, IL-6, and IL-10. (**B**) WBC, NE%, PLT and ESR

As presented in Table 3, serum levels of PCT, IL-6, IL-10, and CRP were significantly elevated in the GN-BSI group compared to the GP-BSI group, while no significant statistical difference was observed in serum levels of ESR, WBC, NE%, and PLT between these two groups. ROC analysis (Fig. 2; Table 4) demonstrated that PCT, IL-6, and IL-10 (AUC > 0.7) were effective biomarkers for distinguishing GP-BSI from GN-BSI, whereas CRP, ESR, WBC, NE%, and PLT exhibited lower efficiency in diagnosing GP-BSI (AUC < 0.60). Optimal cut-off value analysis revealed that PCT, IL-6, and IL-10 showed high diagnostic power for GP-BSI (p < 0.0001, sensitivity > 74%, specificity > 63%), while CRP had lower diagnostic efficiency (p = 0.0064, sensitivity 55.34%, specificity 62.07%) compared to PCT, IL-6, and IL-10 but displayed higher diagnostic efficacy compared to other biomarkers (WBC, NE%, PLT, and ESR) in diagnosing GP-BSI.

**Table 3:** Inflammatory biomarker serum levels in patients with GN-BSI and GP-BSI

Variable	GN-BSI	GP-BSI	*P* value
CRP (mg/L), median (IQR)	132.2 (64.97,226.6)	89.42 (47.84, 183.0)	0.0077
PCT (ng/ml), median (IQR)	11.29 (1.80, 41.63)	0.53 (0.12, 2.94)	< 0.0001
IL-6 (pg/ml), median (IQR)	397.4 (125.1, 1447.0)	172.4 (84.81, 216.1)	0.0003
IL-10 (pg/ml), median (IQR)	67.78 (28.71, 188.4)	5.75 (2.38, 19.35)	0.0105
ESR (cm), median (IQR)	81.0 (58.0, 105.0)	70.0 (50.0, 95.0)	0.0621
WBC (×10^9^/L), median (IQR)	9.51(7.13, 12.82)	9.27 (7.05, 12.84)	0.9082
NE% (%), median (IQR)	83.30 (76.90, 87.70)	80.55 (74.95, 86.43)	0.1092
PLT(×10^9^/L), median (IQR)	167.0 (111.0, 244.0)	169.5 (120.0, 243.3)	0.5366

CRP, C-reactive protein; PCT, procalcitonin; IL-6, interleukin-6; IL-10, interleukin-10; ESR, erythrocyte sedimentation rate; WBC, white blood cell count; NE%, neutrophil percentage; PLT, platelet count; IQR, interquartile range

**Table 4:** Performances of inflammatory biomarkers in GP-BSI identification

Variable	CRP	PCT	IL-6	IL-10	ESR	WBC	NE %	PLT
AUC	0.5894	0.8277	0.7348	0.8592	0.5734	0.5096	0.5521	0.5195
*P* value	0.0064	< 0.0001	< 0.0001	< 0.0001	< 0.0278	0.7317	0.0618	0.484
Cut off	< 97.37	< 3.74	< 245.4	< 18.28	< 73.50	< 7.94	< 82.65	> 159.5
Sensitivity (%)	55.34	86.41	82.05	74.36	57.01	40.74	58.02	57.41
Specificity (%)	62.07	67.44	63.11	82.52	58.5	67.4	54.23	46.39
PPV (%)	27.54	51.45	45.71	61.7	36.75	38.82	39.17	36.67
NPV (%)	77.65	92.55	90.28	89.47	76.29	69.13	71.78	70.14

AUC, Area under the receiver operating characteristic curve; PPV, positive predictive value; NPV, negative predictive value

Furthermore, we analyzed the diagnostic power of PCT, IL-6, IL-10, and CRP in identifying specific bacterial species. As illustrated in Table S4, in the GN-BSI group, no statistically significant difference was found in the serum levels of PCT, IL-6, IL-10, and CRP among infections caused by *Escherichia coli*, *Klebsiella pneumoniae*, *Pseudomonas aeruginosa*, *Enterobacter cloacae* complex, and *Proteus mirabilis*. Similarly, in the GP-BSI group, no statistically significant difference was observed in serum levels of PCT, IL-6, IL-10, and CRP among infections caused by *Staphylococcus aureus*, *Staphylococcus hominis*, *Enterococcus faecalis*, *Enterococcus faecium*, and *Streptococcus* spp (Table S5). Thus, it is evident that PCT, IL-6, and IL-10 play critical roles in diagnosing BSI and identifying pathogen classifications (G+/G-), but have limited ability to differentiate specific bacterial species.

### CRP, PCT, and IL-10 possess diagnostic value in the detection of VRE

Multidrug-resistant organisms (MDROs), including carbapenem-resistant *Enterobacteriaceae* (CRE), carbapenem-resistant *Pseudomonas aeruginosa* (CRPA), vancomycin-resistant *Enterococcus* (VRE), and methicillin-resistant S*taphylococcus aureus* (MRSA), have become a major public health concern due to their resistance to treatment, leading to treatment failure, poor prognosis, and increased mortality rates. Early identification of MDROs is essential to curb their spread. In this study, we assessed the diagnostic performance of CRP, PCT, IL-6, and IL-10 in detecting CRE, CRPA, VRE, and MRSA (as shown in Fig. S2 and summarized in Table 5). Regrettably, these inflammatory biomarkers demonstrated restricted diagnostic effectiveness for CRE, CRPA, and MRSA, with AUC values around 0.6. Nevertheless, when it came to VRE detection, CRP, PCT, and IL-10 exhibited encouraging diagnostic significance, with AUC values of 0.73, 0.75, and 0.75, respectively, as detailed in Table 5. Conversely, IL-6 did not display significant diagnostic significance for VRE, with an AUC value of 0.57. To summarize, CRP, PCT, and IL-10 are valuable diagnostic markers for identifying VRE, while their diagnostic efficacy for other MDROs is relatively limited.

**Table 5:** Comparison of serum levels of inflammatory biomarkers and AUC between MDRO and NMDRO

Variable	CRP (mg/L)			PCT (ng/ml)			IL-6 (pg/ml)			IL-10 (pg/ml)		
	median (IQR)	*P* value	AUC	median (IQR)	*P* value	AUC	median (IQR)	*P* value	AUC	median (IQR)	*P* value	AUC
CRE	134.80 (77.77,210.90)	0.68	0.53	12.77 (2.45,58.80)	0.73	0.50	397.80 (275.20,4633.00)	0.09	0.60	66.09 (35.93,202.30)	0.93	0.51
CSE	126.00 (61.67,222.40)			10.61 (1.72,39.85)			397.40 (119.00,1399.00)			67.78 (21.19,188.40)		
CRPA	48.66 (22.77,290.70)	0.89	0.51	18.75 (7.34,79.18)	0.55	0.60	728.40 (184.60,2274.00)	0.74	0.67	53.14 (21.36,571.6)	0.4	0.57
CSPA	45.99 (19.34,294.40)			13.34 (3.62,50.86)			469.70 (96.13,1650.00)			63.84 (13.71,213.00)		
MRSA	88.00 (41.09,199.40)	0.98	0.51	0.86 (0.20,6.53)	0.08	0.61	127.40 (60.37,209.50)	0.94	0.56	5.55 (1.30,16.92)	0.68	0.57
MSSA	94.00 (52.20,192.70)			0.46 (0.14,1.65)			180.70 (94.79,236.80)			5.88 (2.15,18.74)		
VRE	133.90 (75.11,261.10)	0.23	0.73	2.49 (0.55,27.96)	0.03	0.75	181.40 (101.50,243.60)	0.6	0.57	22.01 (8.64,59.76)	0.06	0.75
VSE	69.45 (29.49,150.20)			0.48 (0.12,2.28)			164.00 (103.40,203.60)			65.05 (5.21,19.53)		

MDRO, multidrug resistant organism; NMDRO, non- multidrug resistant organism; CRE, carbapenem resistant enterobacteriacea; CSE, carbapenem susceptible enterobacteriacea; CRPA, carbapenem resistant *pseudomonas aeruginosa*; CSPA, carbapenem susceptible *pseudomonas aeruginosa*; MRSA, methicillin resistant *staphylococcus aureus*; MSSA, methicillin susceptible *staphylococcus aureus*; VRE, vancomycin resistant enterococcus; VSE, vancomycin susceptible enterococcus

### PCT, IL-6, and IL-10 are useful markers for distinguishing pathogen classifications in BSI mouse models

**Fig. 3:**
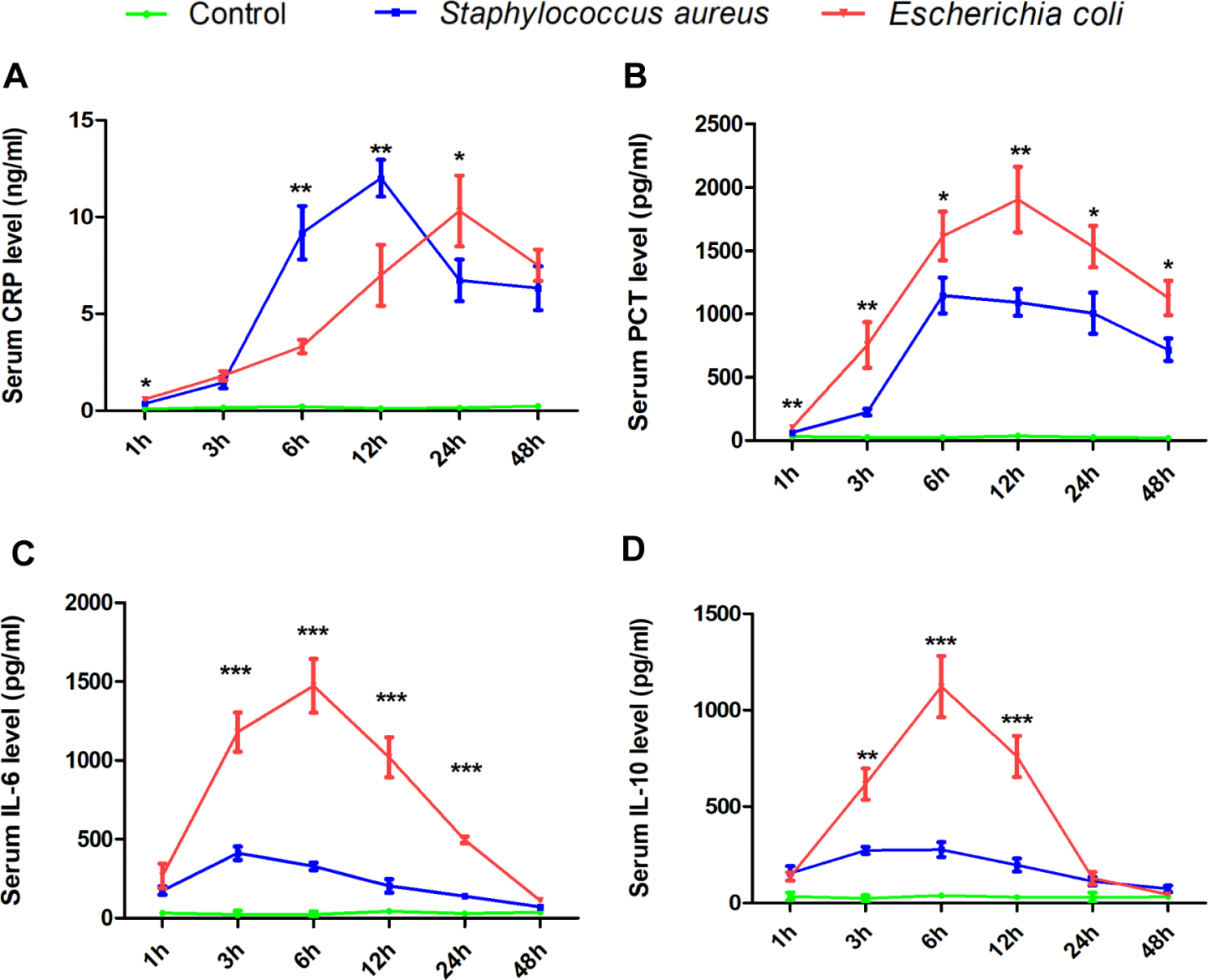
Inflammatory biomarkers expression levels in BSI models at different time points (1 h, 3 h, 6 h, 12 h, 24th, 48 h). (**A**) Analysis of the expression level of CRP. (**B**) Analysis of the expression level of PCT, (**C**) Analysis of the expression level of IL-6, (**D**) Analysis of the expression level of IL-10

Clinical data can be influenced by various factors, such as disease progression, individual differences, environmental factors, and medical interventions. To exclude the impact of these uncertain factors and validate the accuracy of our results obtained from the retrospective clinical study, we utilized two BSI mouse models (GP-BSI and GN-BSI models) in the second phase of our research. These models were established by tail vein injection with a standard strain of ATCC25923 *Staphylococcus aureus* (Gram-positive bacteria) and a standard strain of ATCC25922 *Escherichia coli* (Gram-negative bacteria), respectively. Serum were collected at different time points after bacteria injection to assess serum levels of CRP, PCT, IL-6, and IL-10 in these mouse models. As depicted in Fig. 3, serum CRP levels in both models exhibited a significant increase within the first 3 h after injection of *Staphylococcus aureus* and *Escherichia coli* compared to the control, with no significant difference observed between these two groups at this time point. From the third to the twelfth hour after bacteria injection, serum CRP levels in the GP-BSI model were significantly higher than those in the GN-BSI model. However, after 24 h of bacteria infection, the serum CRP level peaked in the GN-BSI group, surpassing that in the GP-BSI group. These results indicate that serum CRP levels vary at different time points during BSI, with a rapid response to Gram-positive bacteria infection. This suggests that CRP is expessed immediately after Gram-positive bacteria infection and plays a critical role in the early stage of such infections (Fig. 3A).

Interestingly, unlike CRP, serum levels of PCT, IL-6, and IL-10 in the GN-BSI group were consistently higher than those in the GP-BSI group at all time points during 48 h post-infection (Fig. 3B-D). Particularly, IL-6 and IL-10 in the GN-BSI group were significantly higher than those in the GP-BSI group within the first 24 h post-infection. Moreover, serum IL-6 and IL-10 in the GN-BSI group sharply increased within 6 h post-infection, reaching peak levels around 6 h, and then gradually decreased to their original levels within 48 h. These findings demonstrate that PCT, IL-6, and IL-10 are more effective than CRP in differentiating GN-BSI from GP-BSI.

## Discussion

BSI continues to pose a significant global health challenge, resulting in the deaths of approximately eleven million individuals annually. Its impact is particularly pronounced among vulnerable groups such as infants, the elderly, pregnant women, and those residing in low-income countries [24, 25]. Recent research findings indicate that BSI now accounts for one out of every five global deaths, marking a twofold increase in the mortality rate compared to earlier approximations [26]. This ongoing complexity presents substantial obstacles in effectively addressing BSI. The guideline for early goal-directed treatment of BSI emphasizes the importance of early interventions with standard therapies [27]. It underscores the necessity of administering antibiotic treatment within one hour of diagnosis for severe infections, and within four hours for mild infections [27]. Consequently, timely initiation of antibiotic therapy plays a critical role in determining the prognosis for BSI. However, the improper use of antibiotics can contribute to the emergence of challenging multidrug-resistant organisms [28]. Achieving an early and accurate diagnosis, along with precise pathogen classification, stands as the most efficacious approach to treating BSI with appropriate antibiotic strategies and diminishing the risk of multidrug-resistant bacterial strains [27]. BSI biomarkers serve as valuable diagnostic tools, enabling clinicians to quickly identify and treat patients with appropriate measures [29].

The innate immune response is promptly activated upon encountering foreign bodies or antigens. For example, in the case of a gram-negative bacterial infection, toll-like receptors 4 (TLR4) is activated by lipopolysaccharide (LPS) molecules on the bacteria’s surface [30]. This activation leads to the formation of a complex between TLR4, CD14, and MD2, triggering intracellular signaling that results in the production of transcription factors, mainly NF-κB, Ap-1, and IRF3 [31, 32]. Specific cells equipped with pathogen detection components, such as endothelial cells, dendritic cells, natural killer cells, and monocytes, release various inflammatory mediators, including IL-1β, IL-2, IL-6, TNF-α, IL-10, PCT, CRP and others [9]. Therefore, a comprehensive understanding of the biological attributes and pathophysiological roles of diverse cytokines and immune cells proves advantageous in the accurate diagnosis and effective management of infectious diseases.

There are several notable findings in our study. First, our retrospective analysis highlighted striking disparities in the serum levels of these biomarkers between patients afflicted with BSI and LBI, suggesting the potential utility of these biomarkers in differentiating between BSI and LBI cases. Secondly, our investigation underscored the robust diagnostic capability of PCT, IL-6, and IL-10 in distinguishing between GN-BSI and GP-BSI. Conversely, CRP, WBC, NE%, PLT, and ESR demonstrated relatively lower diagnostic efficacy in this context. This points towards the promising role of PCT, IL-6, and IL-10 as biomarkers that can guide clinicians in initiating empirical antibiotic treatments for individuals with BSI. Finally, our study delved into the expression discrepancies of PCT, IL-6, and IL-10 within murine bloodstream infection models, effectively distinguishing between GP-BSI and GN-BSI. In addition, our research outcomes extend to the realm of vancomycin-resistant *Enterococcus*, implicating CRP, PCT, and IL-10 as potential diagnostic indicators.

CRP stands as one of the most widely employed inflammation biomarkers, reflecting the intensity of inflammation within the initial 1 to 3 days following infection [33]. In this study, our dataset validated the elevation of CRP levels in BSI patients compared to those with LBI. Notably, our findings established that CRP could serve as a robust biomarker for diagnosing BSI, as evidenced by its AUC of 0.8149. By employing an optimal CRP threshold of > 68.0 mg/L, we achieved a specificity of 79.41% and a sensitivity of 71.33% in distinguishing BSI from LBI cases. However, it is worth noting that CRP demonstrated limited diagnostic efficacy in discerning between bacterial classifications (G-/G+). These results collectively emphasize the efficacy of CRP as a valuable serum biomarker for BSI diagnosis.

PCT, a member of the calcitonin superfamily of peptides, consists of 116 amino acids and possesses a molecular weight of approximately 14.5 kDa. In the context of infection, heightened expression of the CALC-1 gene amplifies the widespread release of PCT from non-endocrine tissues [34]. Serum levels of PCT are not only significantly elevated in severe systemic infections such as sepsis, septic shock, and severe pneumonia caused by bacterial infection, but also in non-infectious systemic inflammatory syndromes such as surgery and major trauma [35, 36]. Mounting evidence underscores the strong connection between elevated PCT levels and the intensity of inflammation and organ malfunction. This correlation suggests that PCT plays a crucial role in predicting the prognosis of BSI [37]. Yet, only limited research has delved into the potential of PCT in differentiating between systemic infections (e.g., BSI) and localized infections (e.g., pneumonia). A recent meta-analysis indicates that PCT lacks the capability to distinguish sepsis from other non-infectious systemic inflammatory response syndromes in critically ill adults. This suggests that utilizing PCT for patients admitted to critical care units is not advisable [38]. However, our findings demonstrate that PCT serves as a promising biomarker for discerning BSI from LBI. With a defined threshold of PCT > 0.675 ng/ml, the AUC for diagnosing BSI reached 0.8835, yielding a sensitivity of 73.13% and specificity of 87.25%. Moreover, PCT exhibited the ability to not only differentiate BSI from LBI, but also discriminate between G + bacterial infections and G- bacterial infections within the BSI category. When the PCT threshold was set at less than 3.72 ng/ml, the AUC for diagnosing G + bacterial infections was 0.8277, with a sensitivity of 86.41% and specificity of 67.44%. These results strongly indicate that PCT stands as an exceptional biomarker worthy of inclusion in BSI guidelines to facilitate early initiation of antibiotic treatments.

IL-6, categorized as a proinflammatory cytokine, plays a crucial role in the immune system’s response to a range of stimuli such as infections, injuries, and various diseases [39]. It is manufactured by diverse cell types, including T helper 2 (Th2) cells, macrophages, fibroblasts, and endothelial cells, acting as a mediator in immune reactions and aiding in the regulation of inflammation [39]. Conversely, IL-10, identified as an anti-inflammatory cytokine, also holds a pivotal position in immune modulation. It is synthesized by various immune cells, including T cells, macrophages, dendritic cells, and regulatory T cells, which serves as a crucial negative regulator, inhibiting the action of proinflammatory cytokines like IFN-γ and IL-6 [40]. Under pathological circumstances, monocytes exhibit a reduced capacity to release inflammatory mediators like TNF, IL-1, IL-6, and IL-12 upon encountering severe infections. However, their ability to release inhibitory cytokines, particularly IL-10, remains intact and may even be heightened in specific instances [41]. Research studies have provided substantiation that inhibiting IL-10 can mitigate immune suppression and enhance the survival rate of septic mice [42]. Our findings further underscore that patients with severe infections, characterized by heightened levels of PCT and CRP, manifest escalated serum concentrations of IL-10. A previous study has highlighted the potential of IL-6 and IL-10 as markers to differentiate between G- and G + bacterial infections in children with hematological disorders who have BSI [41]. Nonetheless, limited research delves into the diagnostic utility of IL-6 and IL-10 in adult patients with bacterial infections. Our investigation reveals that IL-6 and IL-10 not only distinguish between BSI and LBI but also predict the classifications of potential pathogens. This suggests that serum levels of IL-6 and IL-10 can rapidly discern between G + and G- bacterial infections, thereby offering valuable guidance to clinicians for prompt and suitable administration of antibiotic therapy. Notably, our BSI mouse models have further fortified these observations by demonstrating a rapid upsurge in serum IL-6 and IL-10 levels at the inception of infection, reaching their zenith within the initial 6–12 h, and gradually subsiding to normal physiological levels within 48 h following infection.

Besides inflammatory molecules, the roles of cells involved in the innate immune response are also evident in bacterial infections. Parameters such as PLT, WBC, NE%, and ESR are commonly employed indicators in the diagnosis of BSI [43]. Platelets, which originate from small, disc-shaped cells within the megakaryocyte lineage, play a multifaceted role [44]. The dense granules and α granules present in platelets release vital elements such as Ca2+, vWF, fibrinogen, fibronectin, and platelet factor 4, contributing to hemostasis and coagulation. Simultaneously, platelets exhibit features reminiscent of megakaryocytes and actively participate in the realm of inflammation [44]. During BSI, platelets adhere to and activate vascular endothelial cells, precipitating heightened platelet consumption and degradation [45, 46]. Our investigation indicated a notable reduction in PLT levels among patients with BSI as opposed to the LBI group, a phenomenon potentially attributed to escalated platelet consumption and destruction. Certain studies indicate that lipopolysaccharides present in G- bacteria can induce platelet aggregation, leading to a decline in platelet count in patients with GN-BSI when compared to those with GP-BSI [47, 48]. However, our study does not demonstrate statistically significant discrepancies in PLT counts between GN-BSI and GP-BSI cases.

In cellular immunity, WBC and NE% stand out as the most frequently employed biomarkers for assessing the systemic inflammatory response [49]. During the initial phases of BSI, the mature neutrophils stored in reserve are swiftly activated and released into the peripheral bloodstream, contributing to a robust immune reaction [50]. Our investigation has indicated that WBC and NE% can differentiate BSI from LBI, yet they don’t exhibit the capacity to classify bacterial types. Additionally, ESR ranks among the most commonly utilized indicators for infection diagnosis, owing to its straightforward procedure, high reproducibility, and rapid test outcome availability within one hour [51]. However, its accuracy, specificity, and cut-off value are controversial. Some researchers regard ESR as a good inflammatory marker [52], while others doubt its low sensitivity and specificity in infection [23]. Therefore, the value of ESR in diagnosing infection still remains unclear. Our study illustrates that ESR’s efficacy in successfully distinguishing BSI from LBI, boasting an impressive AUC of 0.9249, which underscores ESR’s robust diagnostic capability for BSI. With an optimal threshold of ESR greater than 53.5 mm, its sensitivity and specificity in pinpointing BSI stand at 76.39% and 91.18%, respectively. Notably, its diagnostic effectiveness surpasses that of procalcitonin (PCT), a finding that deviates from other research findings [53, 54]. We posit that this divergence arises from dissimilar inclusion and exclusion criteria. The subjects in our study comprise adult individuals devoid of tumors and hematological disorders. Furthermore, akin to CRP, WBC, and NE%, ESR exhibits limited ability in distinguishing between G- and G + bacteria during BSI.

Taken together, our study has confirmed the significance of various inflammatory biomarkers in differentiating between BSI and LBI. PCT, IL-6, and IL-10 emerge as dependable indicators for discerning the pathogen classifications within BSI cases. Nevertheless, there exist certain limitations in this study as well. Firstly, the study was conducted at a single medical center, potentially introducing variability when extrapolating the results to a broader population. Secondly, even though patients were selected with strict inclusion and exclusion criteria, the inherent nature of a retrospective study still leaves room for false positives and false negatives. Thirdly, the collection of clinical samples was restricted to a single specific time point, neglecting the dynamic progression of infectious diseases over time.

## Conclusion

BSI represents a highly lethal form of infection. Timely detection, preemptive categorization of pathogen types, and prompt administration of antibiotics are paramount in enhancing prognosis and mitigating fatality rates. Our research underscores the practicality and effectiveness of several clinical biomarkers- CRP, PCT, IL-6, IL-10, ESR, and NE% - in facilitating early BSI diagnosis. Moreover, PCT, IL-6, and IL-10 exhibit the capacity to discern BSI pathogen classifications. These insights carry significant utility in guiding healthcare practitioners towards initiating empiric antibiotic treatments for BSI patients, thereby fostering improved clinical outcomes.

### Electronic supplementary material

Below is the link to the electronic supplementary material.

**Supplementary Material 1**: Figure S1. Evaluation of Inflammatory Biomarkers in Predicting Bloodstream Infections

**Supplementary Material 2**: Figure S2. Discriminatory Efficacy of CRP, PCT, IL-6, and IL-10 in Predicting MDRO Presence

**Supplementary Material 3**: Table S1. Demographic and Clinical Characteristics of Patients with BSI and LBI

**Supplementary Material 4**: Table S2. Serum Levels of Inflammatory Biomarkers in Patients with BSI and LBI

**Supplementary Material 5**: Table S3. Performance of Inflammatory Biomarkers in BSI Diagnosis

**Supplementary Material 6**: Table S4. Comparison of Serum Levels of Inflammatory Biomarkers Among Pathogens in GN-BSI

**Supplementary Material 7**: Table S5. Comparison of Serum Levels of Inflammatory Biomarkers Among Pathogens in GP-BSI

## Data Availability

All authors have agreed that the datasets used and analyzed in this study are available from the corresponding author upon reasonable request.

## Abbreviations

CRP: C-reactive protein
PCT: procalcitonin
IL-6: interleukin-6
WBC: white blood cells count
NE%: neutrophil percentage
PLT: platelets count
ESR: erythrocyte sedimentation rate
BSI: bloodstream infection
LBI: local bacterial infection
GP-BSI: BSI caused by Gram-positive bacteria
GN-BSI: BSI caused by Gram-negative bacteria
ROC: receiver operator characteristic
AUC: area under the ROC curve
