# Supplementary material for: PCT, IL-6, and IL-10 facilitate early diagnosis and pathogen classifications in bloodstream infection
Source: Ann Clin Microbiol Antimicrob. 2023 Nov 20;22:103. doi: 10.1186/s12941-023-00653-4 (PMC10662675; doi:10.1186/s12941-023-00653-4)
Supplement: Supplementary file 1 — Supplementary Material 1: Figure S1. Evaluation of Inflammatory Biomarkers in Predicting Bloodstream Infections [file 12941_2023_653_MOESM1_ESM.docx]

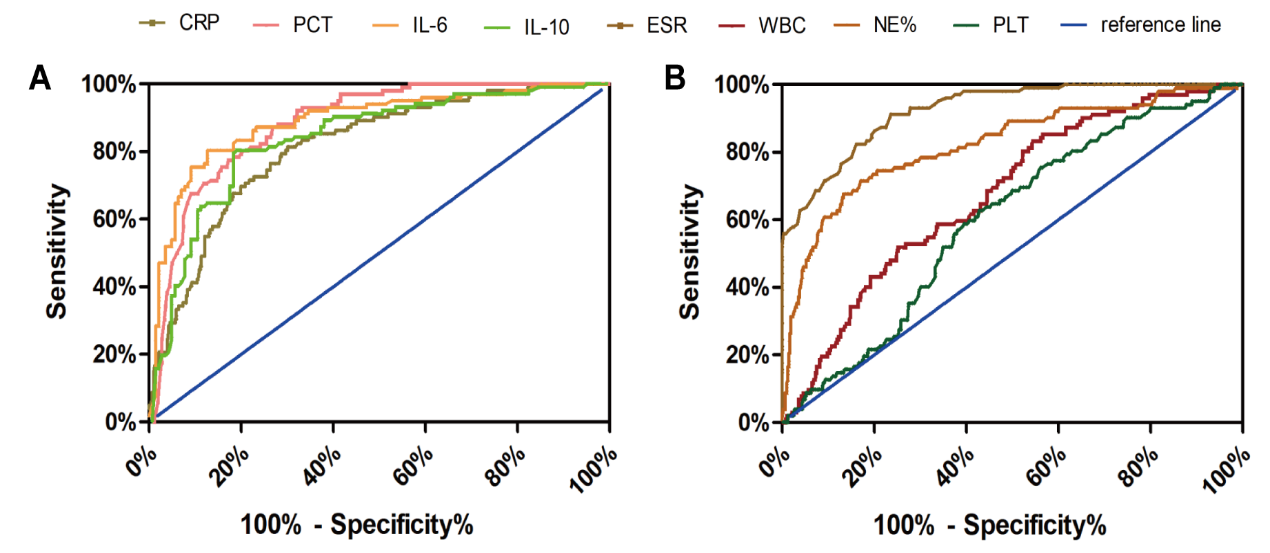


**Fig. S1** Receiver operating characteristic (ROC) curves were used to evaluate the effectiveness of inflammatory biomarkers in predicting bloodstream infections. (A) CRP, PCT, IL-6, and IL-10. (B) WBC, NE%, PLT and ESR. CRP: C-reactive protein, PCT: procalcitonin, IL-6: interleukin-6, IL-10: interleukin-10, WBC: white blood cell count, NE%: neutrophil percentage, PLT: platelet count, ESR: erythrocyte sedimentation rate.
