# Supplementary material for: PCT, IL-6, and IL-10 facilitate early diagnosis and pathogen classifications in bloodstream infection
Source: Ann Clin Microbiol Antimicrob. 2023 Nov 20;22:103. doi: 10.1186/s12941-023-00653-4 (PMC10662675; doi:10.1186/s12941-023-00653-4)
Supplement: Supplementary file 2 — Supplementary Material 2: Figure S2. Discriminatory Efficacy of CRP, PCT, IL-6, and IL-10 in Predicting MDRO Presence [file 12941_2023_653_MOESM2_ESM.docx]

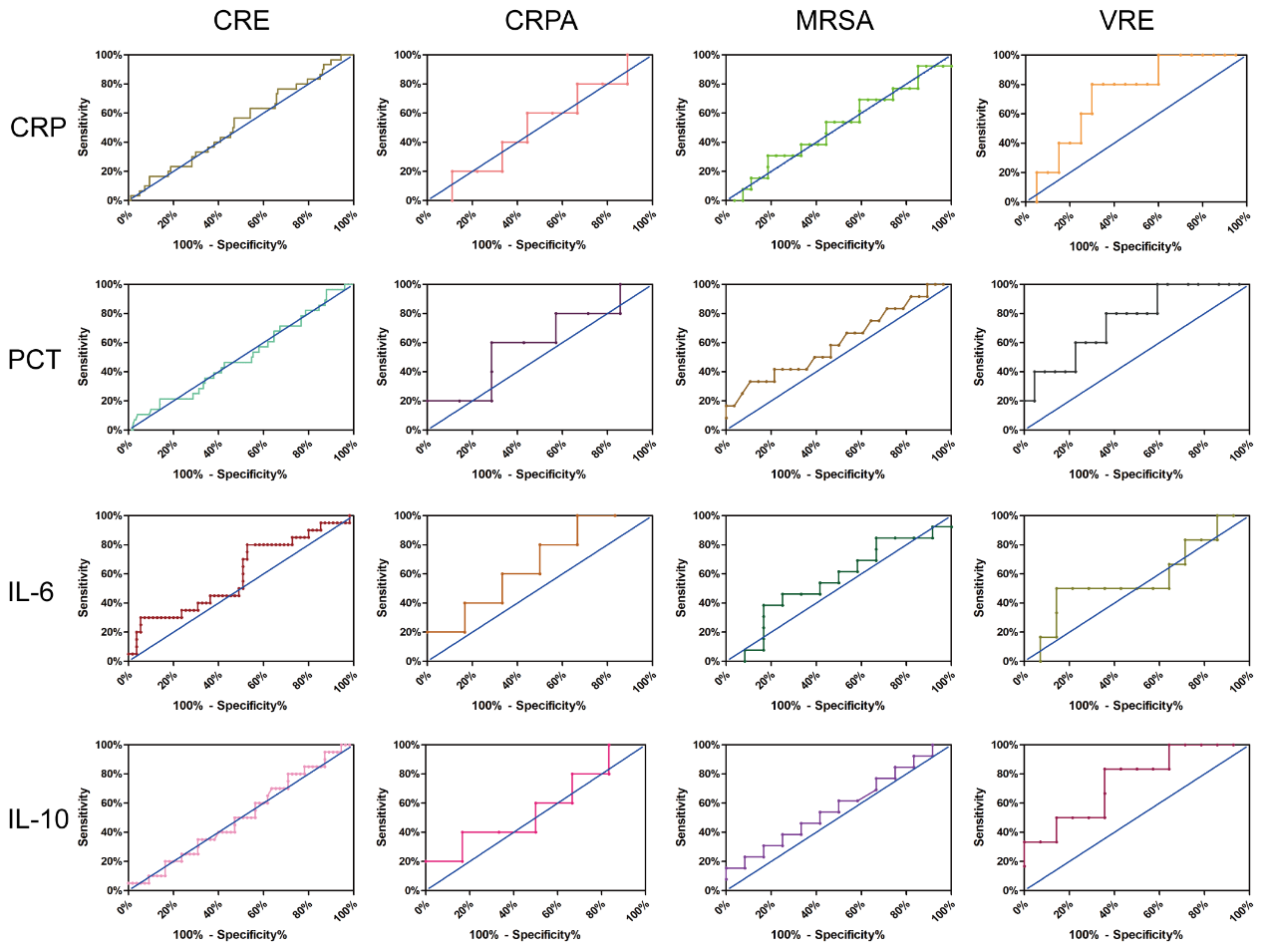


**Fig. S2** Receiver operating characteristic curves were utilized to evaluate the discriminatory efficacy of CRP, PCT, IL-6, and IL-10 in predicting the presence of multidrug-resistant organisms (MDROs). CRE: Carbapenem-Resistant *Enterobacteriaceae*, CRPA: Carbapenem-Resistant *Pseudomonas aeruginosa*, MRSA: Methicillin-Resistant *Staphylococcus aureus*, VRE: Vancomycin-Resistant *Enterococcus*.
