# Supplementary material for: PCT, IL-6, and IL-10 facilitate early diagnosis and pathogen classifications in bloodstream infection
Source: Ann Clin Microbiol Antimicrob. 2023 Nov 20;22:103. doi: 10.1186/s12941-023-00653-4 (PMC10662675; doi:10.1186/s12941-023-00653-4)
Supplement: Supplementary file 3 — Supplementary Material 3: Table S1. Demographic and Clinical Characteristics of Patients with BSI and LBI [file 12941_2023_653_MOESM3_ESM.docx]

**Table S1** The demographic and clinical characteristics of patients with BSI and LBI

| Variable | BSI (n=505) | LBI (n=102) | *P* value |
| --- | --- | --- | --- |
| Age，mean ± SD | 62.60±16.10 | 61.86±19.62 | 0.6835 |
| Gender（male/female） | 267/238 | 52/50 | 0.7272 |
| **Primary diagnoses, n (%)** |  |  |  |
| Diabetes mellitus | 38 (7.52%) | 5 (4.90%) | 0.4056 |
| Traumatic disease | 41 (8.12%) | 9 (8.82%) | 0.8434 |
| Cholecystitis | 31 (6.14%) | 10 (9.80%) | 0.1935 |
| Pneumonia | 43 (8.51%) | 13 (12.75%) | 0.1894 |
| Cardiopathy | 12 (2.38%) | 3 (2.94%) | 0.7264 |
| Peritonitis | 37 (7.33%) | 6 (5.88%) | 0.8321 |
| Urinary tract infection | 45 (8.91%) | 8 (7.84%) | 0.8487 |
| [Appendicitis](javascript:;) | 11 (2.18%) | 4 (3.92%) | 0.2954 |
| Chronic lung disease | 26 (5.15%) | 6 (5.88%) | 0.8075 |
| Hypertension | 16 (3.17%) | 2 (1.96%) | 0.7511 |
| Others | 205 (40.59%) | 36 (35.29%) |  |
| **Clinical** **symptoms, n (%)** |  |  |  |
| Fever | 344 (68.12%) | 47 (46.08%) | <0.0001 |
| Shock | 50 (9.90%) | 7 (6.86%) | 0.4561 |
| Chills | 211 (41.78%) | 25 (24.51%) | 0.0012 |
| **Ward of hospitalization** |  |  |  |
| Medical | 290 (57.43%) | 62 (60.78%) | 0.7093 |
| Surgical | 169 (33.47%) | 33 (32.35%) |  |
| Intensive care unit | 46 (9.11%) | 7 (6.86%) |  |

BSI: bloodstream infection, LBI: local bacterial infection, SD: standard deviation
