# Supplementary material for: PCT, IL-6, and IL-10 facilitate early diagnosis and pathogen classifications in bloodstream infection
Source: Ann Clin Microbiol Antimicrob. 2023 Nov 20;22:103. doi: 10.1186/s12941-023-00653-4 (PMC10662675; doi:10.1186/s12941-023-00653-4)
Supplement: Supplementary file 4 — Supplementary Material 4: Table S2. Serum Levels of Inflammatory Biomarkers in Patients with BSI and LBI [file 12941_2023_653_MOESM4_ESM.docx]

**Table S2** Comparison of serum levels of inflammatory biomarkers between BSI and LBI patients

| Variable | BSI | LBI | *P* value |
| --- | --- | --- | --- |
| CRP (mg/L), median (IQR) | 122.5 (57.87,216.6) | 25.8 (9.75, 61.7) | <0.0001 |
| PCT (ng/ml), median (IQR) | 4.61 (0.545, 32.37) | 0.115(0.0575, 0.29) | <0.0001 |
| IL-6 (pg/ml), median (IQR) | 248.6 (116.8, 904.2) | 23.24 (5.698, 56.15) | <0.0001 |
| IL-10 (pg/ml), median (IQR) | 45.39 (13.89, 154.3) | 2.55 (1.268, 8.225) | 0.008 |
| ESR (cm), median (IQR) | 77.5 (54.0, 102.0) | 18.0 (7.75, 37.0) | <0.0001 |
| WBC (×10^9^/L), median (IQR) | 9.48(7.095, 12.83) | 7.09 (5.653, 9.515) | <0.0001 |
| NE% (%), median (IQR) | 82.70(76.15, 87.50) | 67.85 (60.25, 76.10) | <0.0001 |
| PLT (×10^9^/L), median (IQR) | 168.0 (113.5, 244.0) | 210.5(154.5, 248.3) | 0.0254 |

CRP: C-reactive protein, PCT: procalcitonin, IL-6: interleukin-6, IL-10: interleukin-10, ESR: erythrocyte sedimentation rate, WBC: white blood cell count, NE%: neutrophil percentage, PLT: platelet count, IQR: interquartile range.
