# Supplementary material for: PCT, IL-6, and IL-10 facilitate early diagnosis and pathogen classifications in bloodstream infection
Source: Ann Clin Microbiol Antimicrob. 2023 Nov 20;22:103. doi: 10.1186/s12941-023-00653-4 (PMC10662675; doi:10.1186/s12941-023-00653-4)
Supplement: Supplementary file 5 — Supplementary Material 5: Table S3. Performance of Inflammatory Biomarkers in BSI Diagnosis [file 12941_2023_653_MOESM5_ESM.docx]

**Table S3** Performances of inflammatory biomarkers in BSI diagnosis

| Variable | CRP | PCT | IL-6 | IL-10 | ESR | WBC | NE% | PLT |
| --- | --- | --- | --- | --- | --- | --- | --- | --- |
| AUC | 0.8149 | 0.8835 | 0.8888 | 0.8376 | 0.9249 | 0.6761 | 0.8224 | 0.5992 |
| *P* value | <0.0001 | <0.0001 | <0.0001 | <0.0001 | <0.0001 | <0.0001 | <0.0001 | <0.0017 |
| Cut off | > 68.00 | > 0.6750 | > 82.74 | > 11.23 | > 53.50 | > 7.120 | > 73.30 | < 194.5 |
| Sensitivity (%) | 71.33 | 73.13 | 87.32 | 80.99 | 76.39 | 74.84 | 82.95 | 60.5 |
| Specificity (%) | 79.41 | 87.25 | 80.39 | 80.39 | 91.18 | 51.96 | 71.57 | 58.82 |
| PPV (%) | 93.48 | 95.31 | 86.11 | 85.19 | 96.83 | 88.02 | 93.22 | 87.43 |
| NPV (%) | 40.10 | 47.85 | 82.00 | 75.23 | 52.25 | 30.46 | 47.10 | 24.00 |

AUC: Area under the receiver operating characteristic curve. PPV: positive predictive value, NPV: negative predictive value.
