# Supplementary material for: PCT, IL-6, and IL-10 facilitate early diagnosis and pathogen classifications in bloodstream infection
Source: Ann Clin Microbiol Antimicrob. 2023 Nov 20;22:103. doi: 10.1186/s12941-023-00653-4 (PMC10662675; doi:10.1186/s12941-023-00653-4)
Supplement: Supplementary file 6 — Supplementary Material 6: Table S4. Comparison of Serum Levels of Inflammatory Biomarkers Among Pathogens in GN-BSI [file 12941_2023_653_MOESM6_ESM.docx]

**Table S4** Comparison of serum levels of inflammatory biomarkers among different pathogens isolated from patients with GN-BSI

| Variable | *Escherichia coli* | *Klebsiella pneumoniae* | *Pseudomonas aeruginosa* | *Enterobacter cloacae* complex | *Proteus mirabilis* | *P* value |
| --- | --- | --- | --- | --- | --- | --- |
| CRP (mg/L), median (IQR) | 149.50 (79.98，279.50) | 148.50 (96.57, 251.60) | 98.30 (34.60, 177.90) | 75.56 (54.08,118.90) | 153.30 (74.27, 267.00) | 0.10 |
| PCT (ng/ml), median (IQR) | 13.25 (1.61,62.333) | 19.85 (6.58,60.80) | 4.93 (1.40, 14.33) | 3.67 (1.51, 10.53) | 7.22 (4.96, 12.11) | 0.08 |
| IL-6 (pg/ml), median (IQR) | 445.40 (118.80.20,1392.00) | 1428.00 (318.00,3144.00) | 437.10 (268.00, 1953.00) | 870.70 (119.00,1752.00) | 1238.00 (276.50,2055.00) | 0.07 |
| IL-10 (pg/ml), median (IQR) | 77.05 (26.98, 232.60) | 186.50(66.23, 330.00) | 53.63 (13.60,74.69) | 40.97 (27.71, 57.12) | 50.63 (15.20, 91.87) | 0.36 |
