# Supplementary material for: PCT, IL-6, and IL-10 facilitate early diagnosis and pathogen classifications in bloodstream infection
Source: Ann Clin Microbiol Antimicrob. 2023 Nov 20;22:103. doi: 10.1186/s12941-023-00653-4 (PMC10662675; doi:10.1186/s12941-023-00653-4)
Supplement: Supplementary file 7 — Supplementary Material 7: Table S5. Comparison of Serum Levels of Inflammatory Biomarkers Among Pathogens in GP-BSI [file 12941_2023_653_MOESM7_ESM.docx]

**Table S5** Comparison of serum levels of inflammatory biomarkers among different pathogens isolated from patients with GP-BSI

| Variable | *Staphylococcus aureus* | *Staphylococcus hominis* | *Enterococcus faecalis* | *Enterococcus faecium* | *Streptococcus* spp. | *p* value |
| --- | --- | --- | --- | --- | --- | --- |
| CRP (mg/L), median (IQR) | 126.00 (80.25, 196.50) | 70.85 (54.35, 171.30) | 52.20 (15.20, 89.40) | 63.55 (30.80, 170.80) | 141.70 (43.70, 266.80) | 0.2438 |
| PCT (ng/ml), median (IQR) | 2.68 (0.39, 7.96) | 0.53 (0.11, 1.38) | 0.43 (0.12, 1.69) | 0.80 (0.12, 2.63) | 1.64 (0.64, 3.35) | 0.2264 |
| IL-6 (pg/ml), median (IQR) | 199.90 (60.74, 220.40) | 23.76 (1.98, 172.40) | 130.10 (46.55, 213.60) | 161.80 (123.70, 199.90) | 216.10 (102.40, 472.70) | 0.3092 |
| IL-10 (pg/ml), median (IQR) | 12.04 (1.98, 33.03) | 0.59 (0.59, 5.65) | 9.35 (1.10, 17.59) | 6.06 (5.55, 6.57) | 18.10 (3.17, 116.70) | 0.436 |
